# Infection prevention and control practices of ambulatory veterinarians: A questionnaire study in Finland

**DOI:** 10.1002/vms3.464

**Published:** 2021-03-01

**Authors:** Marie Verkola, Terhi Järvelä, Asko Järvinen, Pikka Jokelainen, Anna‐Maija Virtala, Paula M. Kinnunen, Annamari Heikinheimo

**Affiliations:** ^1^ Faculty of Veterinary Medicine University of Helsinki Helsinki Finland; ^2^ Department of Infectious Diseases Inflammation Center Helsinki University Central Hospital and University of Helsinki HUS Finland; ^3^ Laboratory of Parasitology Department of Bacteria Infectious Disease Preparedness Statens Serum Institut Copenhagen S Denmark; ^4^ Finnish Food Authority Seinäjoki Finland

**Keywords:** hand hygiene, infection control, personal protective equipment, veterinarians, zoonoses

## Abstract

**Background:**

Veterinarians face the risk of contracting zoonotic pathogens. Infection prevention and control (IPC) guidelines stress the importance of proper hand hygiene and personal protective equipment (PPE) to prevent transmission of these pathogens.

**Objectives:**

We aimed to assess how ambulatory livestock and equine veterinarians follow IPC guidelines, when working on farms and in stables.

**Methods:**

We studied hygiene practices of livestock and equine ambulatory veterinarians (*n* = 129) in Finland. A web‐based questionnaire was used to obtain demographic information and information regarding hand‐hygiene facilities and practices, use and cleaning of PPE and cleaning of medical equipment.

**Results:**

According to 66.9% of the respondents, hand‐washing facilities were often adequate on livestock farms, but only 21.4% reported that this was the case in stables (*p* < .001). While 75.0% reported washing their hands or using hand sanitizer always before moving on to the next farm, only 42.5% reported doing this before moving on to the next stables (*p* < .001). Universal protective coat or coverall use was more common in livestock practice than in equine practice (91.6% vs. 27.7%, *p* < .001). Stethoscope cleaning was reported to happen less frequently than once a week by 30.0% of the respondents.

**Conclusions:**

Finnish veterinarians’ self‐reported IPC adherence was far from uniform. IPC was more commonly followed in ambulatory livestock practice perhaps facilitated by better hand‐washing facilities on farms than in stables. The study suggests that education of veterinarians is still needed and that hand‐washing facilities need to be improved even in a high‐income country.

## Graphical abstract

Hand hygiene practices and use of personal protective equipment was studied among ambulatory livestock and equine veterinarians. Adherence to infection prevention and control guidelines was suboptimal. The results indicated poor availability of hand hygiene facilities and use of protective clothing especially in stables and should be noted in veterinary education.

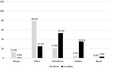

## 1 INTRODUCTION

Zoonotic pathogens are an occupational hazard for veterinarians (Baker & Gray, 2009; Jackson & Villarroel, 2012). Transmission routes of these pathogens to veterinarians include direct contact with colonized or infected animals and contact with a contaminated environment or fomites, such as medical equipment. Infection prevention and control (IPC) practices such as hand hygiene and the use of personal protective equipment (PPE) are an important part of standard procedures to prevent and control health care–associated infections, protect health care providers as well as to reduce the spread of microbes resistant to antimicrobials (WHO, 2019).

Veterinary associations have issued guidelines for the prevention and control of infections and resistant bacteria directed at veterinary practitioners and personnel (Australian Veterinary Association, 2017; BSAVA, 2016; Stull et al., 2018; Williams et al., 2015). Recently, a hygiene guide for companion animal practices and animal hospitals was published in Finland as an output related to the National Action Plan on Antimicrobial Resistance (Thomson & Aaltonen, 2019). Many veterinary practitioners, however, work outside the clinic environment in ambulatory practice, visiting several farms or stables a day. In ambulatory practice, medical equipment and medicines are transported between farms and stables and used at several locations during one day. Facilities for hand hygiene are provided on‐site by the farm or stable owner. Farm owners often provide PPE, mainly coveralls and rubber boots, as part of on‐farm biosecurity for visitors including veterinarians. In veterinary medicine, biosecurity is defined as measures to reduce the risk of diseases entering an animal population, establishing themselves and transmitting within and from the animal population (OIE, 2019). Hand hygiene and PPE for visitors are part of biosecurity guidelines.

In IPC, the goal of non‐surgical hand hygiene is to reduce the amount of transient microbes on the skin (Pittet & Boyce, 2001). While hand washing with plain soap and water removes some of the transient microbes, hand rubbing with alcohol‐based hand rub reduces the bacterial count more effectively and is the fastest and most accessible practice (Allegranzi & Pittet, 2009). Nevertheless, hand washing is an integral part of hand‐hygiene practice, as alcohol‐based hand rubs neither work sufficiently if hands are soiled with organic debris nor on some pathogens, including bacterial spores, non‐enveloped viruses and parasite oocysts. According to recommendations, after wetting hands and applying liquid hand soap, hands should be rubbed for at least 15–20 s before rinsing and drying with disposable paper towels (Centers for Disease Control & Prevention, 2002; Williams et al., 2015). The entire hand‐washing procedure should take 40–60 s (WHO, 2009). When decontaminating clean hands with alcohol‐based hand rub, the procedure should take 20–30 s and hands should be dry at the end of the process (WHO, 2009).

Studies from both human health care settings and veterinary clinic settings have shown poor compliance with hand hygiene (Anderson et al., 2014; Erasmus et al., 2010; Smith et al., 2013; Wright et al., 2008). Most veterinary IPC studies have been conducted among veterinarians working in companion animal clinics or hospitals and mainly focusing on hand‐hygiene practices (Anderson, 2015; Anderson et al., 2014; Espadale et al., 2018). Fewer studies have included veterinarians working in ambulatory livestock or equine practice. Wright et al. (2008) conducted a mail‐out population survey among small animal veterinarians, large animal veterinarians and equine veterinarians in the United States. While 48.4% of small animal veterinarians reported always washing or sanitizing their hands between patient contacts, the proportion of large animal veterinarians and equine veterinarians reporting the same was only 18.2%.

There is room for improvement in both hand hygiene and use of PPE on farms (Nöremark & Sternberg‐Lewerin, 2014; Sahlström et al., 2014). In addition, the level of biosecurity varies—pig farms implement biosecurity more strictly than cattle farms and sheep farms (Nöremark & Sternberg‐Lewerin, 2014; Sahlström et al., 2014). In one study, 75% of veterinarians reported that there were no biosecurity requirements at the stables they visited (Nöremark & Sternberg‐Lewerin, 2014). In the current study, our aim was to assess ambulatory veterinarians’ adherence to general infection control guidelines in Finland. We focused on hand hygiene, use of PPE and cleaning of veterinary equipment used by veterinarians on farms and in stables. We hypothesized that not all veterinarians would comply with hygiene recommendations. In addition, we hypothesized that circumstances conducive to veterinarians’ adherence to proper hand hygiene would be better on farms than in stables.

## 2 MATERIALS AND METHODS

### 2.1 Study design

In 2009, a study on zoonotic infections investigated protection practices of veterinarians in Finland (Kinnunen et al., 2021), and the current study builds on that work. The current study is part of a cross‐sectional study on multi‐resistant bacteria in veterinarians in Finland conducted at the Annual Veterinary Congress in Helsinki, Finland, from 30 November to 2 December 2016 (Verkola et al., 2019). The web‐based questionnaire was open for 3 months, from November 2016 to January 2017 (Data S1). Veterinarians as well as veterinary students authorized to work as veterinarians (after 5 years of studies) were invited to participate in the study.

The web‐based questionnaire was available in Finnish and contained eight sections. Some of the questions were repeated from the study on zoonotic infections in veterinarians conducted in 2009 (Kinnunen et al., 2021). The questionnaire was pretested by 14 veterinarians from different fields and modified according to their feedback prior to the study. Hand washing was defined as rubbing hands with soap and water. As we did not specify the antiseptic agent used for hand sanitation in the original questionnaire, we use ‘hand sanitizer’ in this article. The hand sanitizers used in health care and by veterinarians in the country are usually alcohol based.

In this work, the focus was on ambulatory livestock (farmed animals excluding horses) veterinarians and equine (horse) veterinarians, and on questions about hand hygiene, use of PPE and cleaning of veterinary equipment (Tables S1–S3). Participants were asked to fill in the questionnaire concerning the past 12 months. They were asked to choose the most suitable answer in the questionnaire according to their own assessment and to estimate the time in seconds spent washing their hands.

### 2.2 Statistical analyses

Statistical analyses were carried out using IBM SPSS Statistics versions 24.0 and 25.0 (Armonk, NY, USA). Statistical significance was considered at 0.05 level.

First, frequency tables and cross‐tabulation were utilized. The 95% confidence intervals (CIs) for proportions in our study and in other studies, if they were not reported, were calculated using Wilson's method (Brown et al., 2001) with an Epitools calculator (Sergeant, 2018; http://epitools.ausvet.com.au/content.php?page=CIProportion). Answers of ‘not applicable’ were handled as missing data. Proportions were compared using two‐sample z‐test (https://epitools.ausvet.com.au/ztesttwo), and *p*‐values were adjusted for multiple comparisons by Benjamini‐Hochberg correction (Benjamini & Hochberg, 1995) using the False Discovery Rate (FDR) calculator (https://www.sdmproject.com/utilities/?show=FDR).

In the questionnaire, hand washing and use of hand sanitizer for both between animals or animal groups and before moving on to next farm were asked separately from each other (Table S4). Using the pooled hand washing and hand sanitizer answers, the overall hand hygiene for both between animals or animal groups and before moving on to next farm was calculated, i.e. if the respondent had responded ‘always’ to the question concerning hand washing but ‘often’ to the question concerning hand sanitizer use, the frequency of overall hand hygiene was recorded as ‘always’. If there was no answer for one of the two questions, the response to the other question was used.

The normality of the hand‐washing time estimates was tested with the Shapiro–Wilk test. The association of hand‐washing time frame (<15 s or ≥15 s) with the time of graduation (<10 years ago or ≥10 years ago) was studied using Fisher's exact test.

To assess whether time of graduation, gender or age were associated with the level of IPC practices, an overall precaution awareness (PA) score was calculated (modified from Wright et al., 2008). For this, 20 questions were chosen (Table S5), and the answers based on the points on the Likert scale were summed for each respondent individually. In addition, a separate PA score for livestock practice and another for equine practice were created by summing only the livestock‐related and the general questions, or the equine‐related and the general questions, respectively. A PA score was calculated only for respondents who had answered all the questions included in the calculation. The PA scores were dichotomized into the upper 25% (coded 1) and lower 75% (coded 0). The higher the score, the more stringent the respondent was in IPC practices. Age was classified into three categories with an equal number of respondents in each category. Time of graduation was first classified into three categories and subsequently into four categories. Separate univariable logistic regression analyses were performed for the three categorized PA score variables as dependent variable and ‘time of graduation’ with three categories (not graduated or graduated < 10 years before/graduated 10–20 years before/graduated > 20 years before), ‘time of graduation’ with four categories (not yet graduated/graduated < 10 years before/graduated 10–20 years before/graduated > 20 years before), gender (male/female) and age (up to 31 years/32–43 years/44–65 years) as independent variables. Variables with a *p*‐value < .2 in univariable analyses were entered in the multivariable analysis. To avoid multicollinearity, a phi‐correction coefficient between independent variables was calculated and only variables with *ϕ* < 0.8 were allowed in the same model. In the final analysis, the significance was evaluated based on the *p*‐value < .05. We tested for confounding by including the most potential confounding variable, ‘gender’, into the model and observed its effect on the remaining variables. The confounder was included in the model even if it was not statistically significant if it changed the odds ratio approximately 20%. Two‐way interactions between all variables in the models were also tested.

## 3 RESULTS

### 3.1 Respondents

**FIGURE 1:**
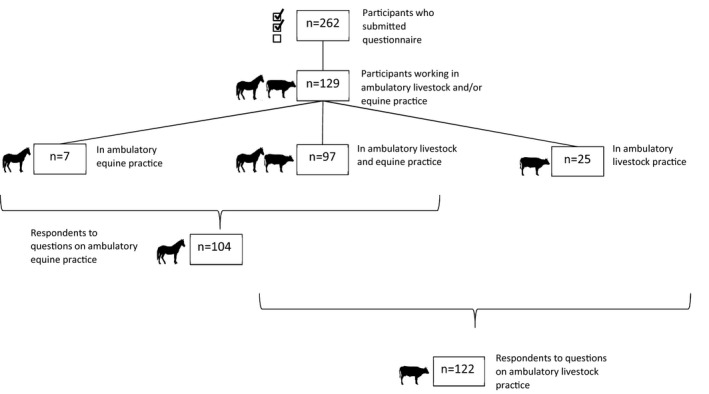
Number of respondents to web‐based questionnaire according to practice type

A total of 262 of approximately 1,000 veterinarians attending the conference answered the questionnaire, representing 10% of all authorized veterinarians (*N* = 2,633, the Registry of Veterinarians, Finnish Food Authority) in Finland. The number of veterinarians working in livestock and/or equine ambulatory practice was 129 (129/262; 49.2%) (Figure 1). These respondents were predominantly women (105/129; 81.4%) (Table 1). Half of the respondents (65/129; 50.4%) had graduated less than 10 years before the time of the study; this included 15 (15/129; 11.6%) who had not graduated yet. Most of the respondents worked both in livestock and equine practice with variable frequency and few worked in only one of those (Table 2). Most (89.9%, i.e. 116/129 veterinarians) worked also in companion animal practice. The number of responses to the individual questions on hygiene measures varied between 101 and 127 mostly because some respondents considered some questions not applicable. The mean number of missing responses per question was 1.4, ranging from 0 to 6.

**TABLE 1:** Background characteristics of veterinarians in the study (*n* = 129)

Category	Subcategory	*n* (%)
Gender	Female	105 (81.4)
	Male	23 (17.8)
	Not disclosed	1 (0.8)
Years since graduation	Not yet graduated	15 (11.7)
	0–9	50 (38.8)
	10–20	31 (24.0)
	>20	33 (25.6)
Location of veterinary education	Helsinki, Finland	116 (89.9)
	Abroad	13 (10.1)

**TABLE 2:** Veterinarians (*n* = 129) working in different types of ambulatory practice. The same veterinarian may work in various types of practice

Type of practice	No of veterinarians	Frequency of practice	Number of veterinarians working in		
			Equine practice only	Livestock^a^ and equine practice	Livestock^a^ practice only
Equine	104				
	36 (35%)	At least weekly	1	35	0
	68 (65%)	Less frequently	6	62	0
Cattle	114				
	71 (62%)	At least weekly	0	62	9
	43 (38%)	Less frequently	0	35	8
Pig	77				
	17 (22%)	At least weekly	0	15	2
	60 (78%)	Less frequently	0	54	6
Poultry	46				
	7 (15%)	At least weekly	0	6	1
	39 (85%)	Less frequently	0	33	6
Fur animal	11				
	0	At least weekly	0	0	0
	11 (100%)	Less frequently	0	11	0
All veterinarians (*n* = 129)			7	97	25

^a^ Livestock practice includes cattle, pig, poultry and/or fur animal practice.

### 3.2 Hand‐washing facilities

**FIGURE 2:**
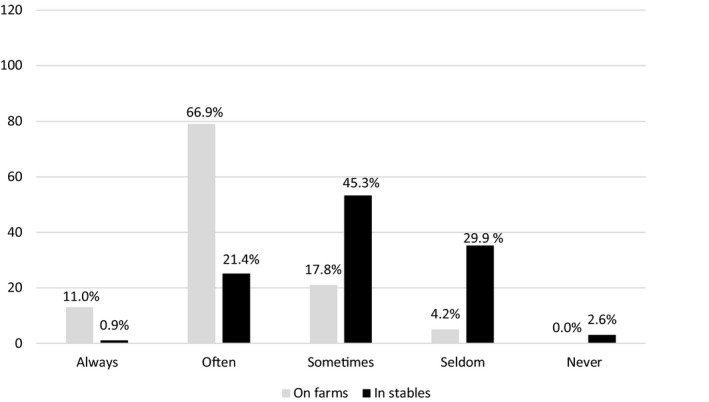
Adequate hand‐washing facilities (warm water, soap, fresh towel/paper hand towels) reported by respondents (*n* = 129). Number of respondents on *y*‐axis and proportions above columns

Altogether, 66.9% of the veterinarians reported that facilities for hand washing were often adequate (defined in questionnaire as warm water, soap, clean towel/paper hand towels) on farms (Figure 2, Table S6). In stables, the corresponding percentage was significantly lower, 21.4% (*p* < .001).

### 3.3 Hand‐hygiene practices

Altogether, 55.1% of the veterinarians reported always washing their hands when they were dirty on farms, while the percentage was 28.0% in stables (*p* < .001) (Table 3). Overall hand‐hygiene compliance including both hand washing or hand sanitizer use was 75.0% reportedly before moving on to the next farm and 42.5% before moving on to the next stable (*p* < .001).

**TABLE 3:** Hand‐hygiene practices in livestock practice and equine practice reported by veterinarians (*n* = 129)

	Livestock practice			Equine practice			Livestock vs. equine practice	
	*n*	%	95% CI	*n*	%	95% CI	*p*‐value^a^	corrected *p*‐value^b^
Washing hands when dirty								
Always*	65	55.1	46.1–63.8	33	28.0	20.7–36.7	<.001	<.001
Often	44	37.3	29.1–46.3	58	49.2	40.3–58.1	.07	.19
Sometimes*	7	5.9	2.9–11.7	21	17.8	11.9–25.7	<.01	.02
Seldom	2	1.7	0.5–6.0	5	4.2	1.8–9.5	.26	.40
Never	0	0.0	0.0–3.2	1	0.8	0.1–4.6	.34	.49
Total	**118**			**118**				
Use of hand sanitizer after washing hands								
Always	7	6.0	2.9–11.8	3	2.5	0.9–7.2	.18	.32
Often	15	12.8	7.9–20.1	24	20.3	14.1–28.5	.12	.28
Sometimes	22	18.8	12.8–26.8	28	23.7	17.0–32.2	.36	.52
Seldom	41	35.0	27.0–44.0	42	35.6	27.5–44.6	.92	.95
Never	32	27.4	20.1–36.1	21	17.8	11.9–25.7	.08	.21
Total	**117**			**118**				
Overall hand hygiene between animals/animal groups								
Always	8	6.8	3.5–12.9	10	8.8	4.8–15.4	.57	.75
Often	40	34.2	26.2–43.2	29	25.4	18.3–34.1	.14	.28
Sometimes	29	24.8	17.8–33.3	36	31.6	23.8–40.6	.25	.40
Seldom	36	30.8	23.1–39.6	30	26.3	19.1–35.1	.45	.63
Never	4	3.4	1.3–8.5	9	7.9	4.2–14.3	.14	.28
Total	**117**			**114**				
Overall hand hygiene before moving on to next farm								
Always*	87	75.0	66.4–82.0	48	42.5	33.8–51.7	<.001	<.001
Often*	25	21.6	15.0–29.9	46	40.7	32.1–49.9	<.01	.01
Sometimes*	3	2.6	0.9–7.3	14	12.4	7.5–19.7	<.01	.02
Seldom	1	0.9	0.2–4.7	3	2.7	0.9–7.5	.30	.46
Never	0	0.0	0.0–3.2	2	1.8	0.5–6.2	.15	.28
Total	**116**			**113**				

Abbreviation: CI, confidence interval.

^a^ z‐test.

^b^ Benjamini‐Hochberg false discovery rate correction.

* Statistically significant at 5% level.

The mean self‐reported hand‐washing time among 112 respondents was 15.2 s (median 10.0 s). After removing two outliers reporting 115 and 150 s, the mean was 13.1 s (median 10.0 s). There was no statistically significant difference between hand‐washing time and years since graduation.

### 3.4 Use of hand sanitizer and protective gloves

Of all the respondents, 27.8% always and 42.1% often used hand sanitizer after doffing protective gloves (Table S7). More than half of the respondents reported always using protective gloves when treating a contaminated, fresh wound in livestock or in horses (Table 4). For both livestock and horses, the same three respondents reportedly never used gloves when treating a contaminated, fresh wound. Three quarters of respondents reported always using gloves when treating a wound showing signs of infection in livestock or in horses. Two respondents reportedly never used gloves when treating an infected wound in livestock and one respondent in horses. There was no statistically significant difference between glove use in livestock practice and in equine practice when treating a contaminated, fresh wound or an infected wound.

**TABLE 4:** Protective gloves and clothes worn when working in livestock practice and equine practice as reported by web‐based questionnaire respondents

	Livestock practice			Equine practice			Livestock vs. equine practice	
	*n*	%	95% CI	*n*	%	95% CI	*p*‐value^a^	Corrected *p*‐value^b^
Use of protective gloves								
Always	11	9.3	5.3–15.9	6	5.1	2.4–10.7	.21	.36
Often*	33	28.0	20.7–36.7	12	10.2	5.9–16.9	<.001	<.01
Sometimes	42	35.6	27.5–44.6	39	33.1	25.2–42.0	.69	.83
Seldom	26	22.0	15.5–30.3	40	33.9	26.0–42.8	.04	.13
Never*	6	5.1	2.4–10.7	21	17.8	11.9–25.7	<.01	.01
Total	**118**			**118**				
Use of protective gloves when treating a contaminated, fresh wound								
Always	64	59.8	50.3–68.6	73	68.9	59.5–76.9	.17	.31
Often	33	30.8	22.9–40.1	22	20.8	14.1–29.4	.10	.23
Sometimes	4	3.7	1.5–9.2	4	3.8	1.5–9.3	.97	.98
Seldom	3	2.8	1.0–7.9	4	3.8	1.5–9.3	.68	.83
Never	3^c^	2.8	1.0–7.9	3^c^	2.8	1.0–8.0	1.00	1.00
Total	**107**			**106**				
Use of protective gloves when treating an infected wound (signs of infection)								
Always	76	72.4	63.2–80.0	80	79.2	70.3–86.0	.26	.40
Often	24	22.9	15.9–31.8	15	14.9	9.2–23.1	.14	.28
Sometimes	2	1.9	0.5–6.7	3	3.0	1.0–8.4	.61	.77
Seldom	1	1.0	0.2–5.2	2	2.0	0.5–6.9	.55	.75
Never	2^c^	1.9	0.5–6.7	1^c^	1.0	0.2–5.4	.59	.77
Total	**105**			**101**				
Wearing protective coat/coveralls								
Always*	109	91.6	85.2–94.4	33	27.7	20.5–36.4	<.001	<.001
Often*	10	8.4	4.6–14.8	33	27.7	20.5–36.4	<.001	<.001
Sometimes*	0	0.0	0.0–3.1	27	22.7	16.1–31.0	<.001	<.001
Seldom*	0	0.0	0.0–3.1	13	10.9	6.5–17.8	<.01	<.01
Never*	0	0.0	0.0–3.1	13	10.9	6.5–17.8	<.01	<.01
Total	**119**			**119**				
Wearing work footwear								
Always*	114	95.8	90.5–98.2	75	63.0	54.1–71.2	<.01	<.01
Often*	5	4.2	1.8–9.5	26	21.8	15.4–30.1	<.01	<.01
Sometimes*	0	0.0	0.0–3.1	10	8.4	4.6–14.8	<.01	<.01
Seldom	0	0.0	0.0–3.1	4	3.4	1.3–8.3	.04	.13
Never	0	0.0	0.0–3.1	4	3.4	1.3–8.3	.04	.13
Total	**119**			**119**				
Wearing headgear								
Always	23	19.7	13.5–27.8	10	8.5	4.7–14.9	.01	.05
Often	28	23.9	17.1–32.4	19	16.1	10.6–23.8	.13	.28
Sometimes	24	20.5	14.2–28.7	25	21.2	14.8–29.4	.89	.95
Seldom	21	17.9	12.0–25.9	22	18.6	12.6–26.6	.89	.95
Never*	21	17.9	12.0–25.9	42	35.6	27.5–44.6	.01	.01
Total	**117**			**118**				

Abbreviation: CI, confidence interval

^a^ z‐test.

^b^ Benjamini‐Hochberg false discovery rate correction.

^c^ These were the same respondents.

* Statistically significant at 5% level.

### 3.5 Use of personal protective equipment

Protective coats or coveralls were used by 91.6% of all the respondents always when working in livestock practice and by 27.7% in equine practice (*p < *.001; Table 4). Work footwear was always worn by 95.8% of the respondents in livestock practice and 63.0% in equine practice (*p* < .01).

### 3.6 Cleaning of equipment

**FIGURE 3:**
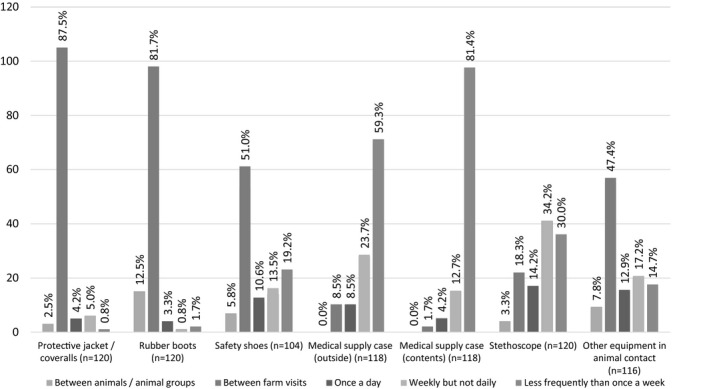
Cleaning or replacement of possible fomites used during farm/stables visits reported by the respondents (*n* = 129). Number of respondents on the *y*‐axis and proportions above columns

Protective coats or coveralls were cleaned or changed between farm visits by 87.5% of the respondents, and rubber boots by 81.7% (Figure 3). Safety shoes were cleaned or changed between visits by 51.0%. The outside of the medical supply case was cleaned by most (59.3%) and its contents by 81.4% less frequently than once a week. Four respondents of 120 (3.3%) reported cleaning their stethoscope between animals or animal groups, 18.3% between farms and 30.0% less frequently than once a week.

### 3.7 Infection control behaviour based on precaution awareness score

The overall PA score was calculable for 86/97 (88.7%) respondents, the PA score for livestock practice for 97/122 (79.5%) respondents and for equine practice for 90/104 (86.5%) respondents (Table 5). From the univariable logistic regression analyses (Table S8), only the four‐category ‘years since graduation’ variable was significantly associated with the overall PA score and the PA score for equine practice, none with the PA‐score livestock practice. Age was highly correlated with years since graduation wherefore they could not be included in the same model. No interactions between variables were detected. Gender changed the odds ratio of the ‘years since graduation’ and was kept to control for confounding. In the following multivariable (or two‐variable) models for overall PA score and PA score for equine practice, those not yet graduated had significantly higher odds for better score (Tables S9–S11). However, their numbers were low.

**TABLE 5:** Questions used for calculation of the overall precaution awareness (PA) score (all questions), PA score for livestock practice (questions on hygiene behaviour in livestock practice and general hygiene behaviour) and PA score for equine practice (questions on hygiene behaviour in equine practice and general hygiene behaviour ), frequency of answer options, median and range on Likert scale. Scale used for questions on hand hygiene and use of personal protective equipment: 0 = never, 1 = seldom, 2 = sometimes, 3 = often, 4 = always; for last three questions on frequency of cleaning: 0 = less frequently than once a week, 1 = weekly but not daily, 2 = once a day, 3 = between farm visits, 4 = between animals/animal groups

	Hygiene behaviour in livestock practice			Hygiene behaviour in equine practice		
(a)						
Question	Frequency of answer options	Median	Range	Frequency of answer options	Median	Range
Washing hands when dirty	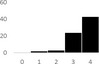	4	1–4	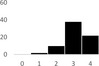	3	1–4
Overall hand hygiene between animals/animal groups	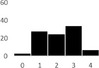	2	0–4	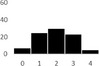	2	0–4
Overall hand hygiene before moving on to the next farm	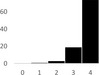	4	1–4	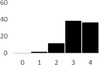	3	1–4
Use of hand sanitizer after washing hands	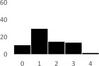	1	0–4	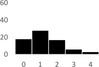	1	0–4
Use of protective gloves when treating a contaminated, fresh wound	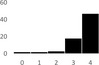	4	0–4	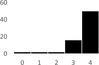	4	0–4
Use of protective gloves when treating infected wound	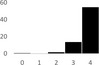	4	0–4	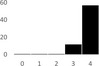	4	0–4
Wearing protective jacket/ coveralls	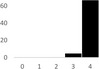	4	3–4	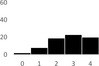	4	0–4
Wearing work footwear	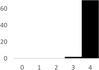	4	3–4	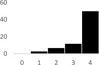	4	1–4

5b.			
General hygiene behaviour			
Question	Frequency of answer options	Median	Range
Use of hand sanitizer after doffing protective gloves	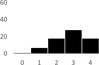	3	0–4
Frequency of cleaning/ changing protective jacket/coveralls	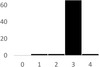	3	1–4
Frequency of cleaning/ changing rubber boots	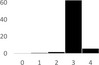	3	1–4
Frequency of cleaning/ changing stethoscope	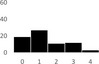	1	0–4

## 4 DISCUSSION

The results of this study add to the knowledge on IPC practices of veterinarians. This study showed that hand‐hygiene facilities were poor in stables and suboptimal on farms, and even the self‐reported hand‐hygiene practices of the veterinarians did not meet recommendations. In addition, veterinarians often failed to use adequate PPE to protect themselves and their patients from transmission of pathogens.

The 262 veterinarians who answered the questionnaire covered 10% of all authorized veterinarians in Finland of which more than 85% belonged to the Finnish Veterinary Association (Finnish Veterinary Association, 2016a). Compared with the age distribution of the members of the association, veterinarians younger than 30 years were slightly over‐represented (Finnish Veterinary Association, personal communication, 24 April 2020). The gender distribution is in line with the female‐dominated veterinary population (Finnish Veterinary Association, 2016b). Three quarters of the respondents worked in both ambulatory livestock practice and equine practice, reflecting well the municipal veterinary system in Finland. Municipal veterinarians treat all animal species and are the largest professional group to treat livestock in Finland.

Both biosecurity and infection control guidelines stress the importance of hand hygiene. For adequate hand washing, water, soap and a clean towel or, ideally, disposable paper hand towels are necessary (WHO, 2009). In this study, 33% of the veterinarians reported that adequate hand‐washing facilities were seldom or never available in stables. This is an even poorer finding than in a Swedish study on biosecurity on farms and in stables (Nöremark & Sternberg‐Lewerin, 2014), where 24% of the veterinarians reported that none or almost none of the stables had hand‐washing facilities available. Availability and accessibility of the facilities is a key factor influencing the frequency of hand washing (Hugonnet & Pittet, 2000).

The poor availability of hand‐washing facilities alone, however, cannot fully explain the poor hand‐hygiene compliance between animals and animal groups and before moving on to the next farm. Hand washing is only necessary when hands are visibly soiled (WHO, 2009). In all other situations, alcohol‐based hand rub is the preferred alternative. Poor availability of hand sanitizer on farms (Suokorpi et al., 2019) and in stables is one likely explanation for lack of hand‐hygiene compliance in our study. Regarding human health care workers’ compliance, easy access has been shown to be important to increase use of hand sanitizer (Traore et al., 2007). It should be noted that to prevent zoonotic transmission of pathogens, hand hygiene should be performed when leaving animal premises and not while stopping for fuel between farms or in the car, as reported in another study (Nöremark & Sternberg‐Lewerin, 2014). Therefore, it is somewhat alarming that poor washing facilities were not compensated by more frequent hand sanitizer use by the veterinarians—a fact that necessitates more education.

In our study, the corrected mean of the self‐reported hand‐washing time was less than the recommended minimum of 15 s. The corrected mean hand‐washing time, 13.1 s, however, was higher than the observed hand‐washing times in companion animal clinics where mean contact time for soap was 4 s (Anderson et al., 2014). One explanation is that self‐reporting resulted in over‐estimation of the actual duration.

Hygiene recommendations for ambulatory veterinary practice are few and are often extrapolated from human health care settings. However, farms and stables are very different environments from hospitals, and livestock and horses may carry higher microbe loads on their skin than an average human patient (Traub‐Dargatz et al., 2006; Weese, 2004). According to one small study, hand rubbing with alcohol‐based hand sanitizer was actually more effective in reducing bacterial loads after routine equine physical examination in a clinic setting than hand washing for 15 s with antiseptic soap (Traub‐Dargatz et al., 2006). However, in the farm and stable environment hands may more easily get extensively soiled with organic debris than while performing a physical examination in a clinic. Therefore, further studies are needed to determine the adequate hand‐hygiene procedures for ambulatory veterinary practice.

In this study, a protective coat or coveralls was used significantly more often in livestock practice than in equine practice. It has been earlier reported that horse owners did not consider protective clothing necessary (Nöremark & Sternberg‐Lewerin, 2014). In our study, it was noteworthy that while rubber boots were cleaned or changed by most veterinarians between farm visits, safety shoes were changed or cleaned only by half. Safety shoes are mostly worn in equine practice, while rubber boots are worn on farms. Thus, work footwear appears to be another point where infection control is better implemented in livestock practice than in equine practice. Reasons for veterinarians not using protective clothing in equine practice might be low zoonotic risk perception or lack of official biosecurity requirements due to the different nature of the livestock and equine sectors. While the food industry and livestock producers’ association Animal Health ETT have been promoting and instructing biosecurity on Finnish farms since the 1990s, there are no national biosecurity guidelines for the equine sector.

Stethoscopes have acted as fomites in outbreaks in human health care facilities (Kanamori et al., 2017). In this study, the stethoscope was reportedly rarely cleaned between animals or animal groups and between farms. Similar findings have been reported from small animal veterinary hospitals and a veterinary teaching hospital (Fujita et al., 2013; KuKanich et al., 2012). The studies showed frequent bacterial contamination of the diaphragms of the stethoscopes with potentially pathogenic and zoonotic, both susceptible and resistant *Staphylococcus* spp., *E. coli* and *Enterococcus* spp. strains. Enterococcal growth was shown on rectal thermometers as well (KuKanich et al., 2012). The rectal thermometer and the ultrasound probe have also been identified as fomites in health care facilities (Kanamori et al., 2017). To fight against the spread of pathogens, including zoonotic and possibly antimicrobial‐resistant microbes, the awareness of veterinarians and others moving between farms may be of pivotal importance. This should be one future aspect in continued education.

Analysing the PA scores we found that respondents who had not yet graduated had higher odds to have an overall PA score and a PA score for equine practice in the upper 25% than respondents who had graduated over 20 years ago. Respondents who had graduated less than 10 years earlier did not have higher odds than respondents who had graduated over 20 years ago. As hygiene education in veterinary education has not changed substantially during the last decade, it is possible that either students were more likely to give responses that they thought were expected of them or veterinarians tend to get more lax in their hygiene practices during the years following graduation. It should be noted, however, that the number of veterinarians not yet graduated included in the models was very small.

There are further limitations to this study. The answer options in the questionnaire ‘always’, ‘often’, ‘sometimes’, ‘seldom’ and ‘never’ were not defined accurately and are likely to have been perceived differently by the individual respondents. The same applies to other terms used in the questionnaire such as ‘dirty’ hands. The term ‘visibly soiled’ used by the WHO should have been adopted for the questionnaire, even though it has some problems from a transcultural perspective (WHO, 2009). Another important limitation of this study is that it is based on self‐reporting and response bias is likely. Especially social‐desirability bias is common in hand‐hygiene studies based on self‐reported data resulting in over‐estimation of compliance (Contzen et al., 2015). Self‐reported hand‐washing time is particularly unreliable and observational studies should be employed to verify these findings. Furthermore, hygiene behaviour between animals and animal groups and when moving on to the next farm was enquired separately for hand washing and hand sanitizer use, although in most cases either method is appropriate. Therefore, the results of both questions were combined for the analyses. The more optimal response of the two responses was chosen for the overall hand‐hygiene behaviour, but this does not take into account that a respondent might, for instance, have chosen ‘always’ for the overall question instead of ‘sometimes’ and ‘often’ for the individual questions.

It may neither be possible nor reasonable to uphold a similar biosecurity level in stables to that of farms due to the regular traffic of horses and horse owners or clients (Weese, 2014). However, this is no reason for negligence. Hand hygiene is highly effective in infection control, and providing proper facilities for hand washing as well as antiseptic hand rub is a cost‐effective solution to improve the situation.

## 5 CONCLUSIONS

This study indicates that even self‐reported IPC adherence of Finnish veterinarians is far from adequate. Compliance to IPC guidelines was more common in ambulatory livestock practice than equine practice. This may partly be due to better hand‐washing facilities on farms as compared with stables. The study suggests that further education is needed and that even in a high‐income country hand‐washing facilities on farms and in stables need improvement.

## 6 ETHICS STATEMENT

All participants provided written informed consent. The study was approved by the Coordinating Ethics Committee of the Hospital District of Helsinki and Uusimaa (HUS/1446/2016).

## CONFLICTS OF INTEREST

PMK is affiliated to MSD Animal Health. This study was completed before joining the company, and MSD Animal Health has not had any influence on the content of this article.

## AUTHOR CONTRIBUTION

**Marie Verkola:** Data curation; Formal analysis; Investigation; Visualization; Writing‐original draft. **Terhi Järvelä:** Data curation; Formal analysis; Writing‐original draft. **Asko Järvinen:** Conceptualization; Writing‐review & editing. **Pikka Jokelainen:** Conceptualization; Writing‐review & editing. **Anna‐Maija K. Virtala:** Formal analysis; Methodology; Writing‐review & editing. **Paula M. Kinnunen:** Conceptualization; Funding acquisition; Writing‐review & editing. **Annamari Heikinheimo:** Conceptualization; Funding acquisition; Investigation; Methodology; Project administration; Resources; Supervision; Validation; Writing‐review & editing.

### PEER REVIEW

The peer review history for this article is available at https://publons.com/publon/10.1002/vms3.464.

## Supporting information

Table S1‐S11

Supplementary Material

## Data Availability

The data that support the findings of this study are available on request from the corresponding author. The data are not publicly available due to privacy or ethical restrictions.
