# Supplementary material for: Infection prevention and control practices of ambulatory veterinarians: A questionnaire study in Finland
Source: Vet Med Sci. 2021 Mar 1;7(4):1059–70. doi: 10.1002/vms3.464 (PMC8294370; doi:10.1002/vms3.464)
Supplement: Supplementary file 1 — Table S1‐S11 [file VMS3-7-1059-s002.pdf]

TABLE S1 Questions in questionnaire section ‘Veterinary qualification and experience’ which were analysed in this study.

Question	Answer options
Gender	Male/Female/Other
Year of birth	Chosen from list of answer options
Occupation	Substitute/Municipal veterinarian/Practitioner as an employee/Entrepreneur/Government or European Union/Employee in industry/Student/Retired/ Temporarily absent from work
Location of veterinary education	Finland/Abroad
Graduation year	Chosen from list of answer options

TABLE S2. Questions in questionnaire section ‘Occupational conditions and exposure to animals, cadavers, carcasses and animal origin specimen’ which were analysed in this study.

Categories involved/section of interest	Answer options
Contact with:	Yes/No to all
Live animals	
Carcasses or cadavers	
Specimens of animal origin	
Distribution of work:	At least weekly/Less frequently/Not at all to all
Cattle practice	
Pig practice	
Poultry practice	
Fur animal practice	
Ambulatory equine practice	
Companion animals in practice/clinic	
Companion animals in hospital	

TABLE S3 Questions in questionnaire section ‘Occupational risk factors and protective measures’ which were analysed in this study

Categories involved	Answer options
Hand-washing practices:	Always/Often/Sometimes/Seldom/Never to all
Adequacy of conditions for hand-washing (warm water, soap, fresh towel/paper hand towels)	
Washing hands when dirty	
Washing hands between animals/animal groups	

Washing hands before moving on to next farm	
Use of hand sanitizer[a]:<br>Use of hand sanitizer after washing hands<br>Use of hand sanitizer between animals/animal groups<br>Use of hand sanitizer before moving on to next farm<br>Use of hand sanitizer after doffing protective gloves	Always/Often/Sometimes/Seldom/Never to all
Time used to wash hands	Open-ended
Use of protective gear[a]:<br>Use of protective gloves in livestock practice<br>Use of protective gloves in equine practice<br>Use of protective gloves when treating a contaminated, fresh [illegible]<br>Use of protective gloves when treating a contaminated, fresh wound in horses<br>Use of protective gloves when treating an infected wound (signs of infection) in livestock<br>Use of protective gloves when treating an infected wound (signs of infection) in horses<br>Wearing protective jacket/coveralls in livestock practice<br>Wearing protective jacket/coveralls in equine practice<br>Wearing work footwear in livestock practice<br>Wearing work footwear in equine practice<br>Wearing headgear in livestock practice<br>Wearing headgear in equine practice	Always/Often/Sometimes/Seldom/Never to all
Frequency of cleaning/changing equipment:<br>Protective jacket/coveralls<br>Rubber boots<br>Safety shoes	Between animals or animal groups/Between farm visits/ Daily/Weekly but not daily/Less frequently than once a week to all

Outside of medical supply case
Contents of medical supply case
Stethoscope
Other equipment in animal contact

[a]when working with live animals

TABLE S4 Hand-washing and hand sanitizer use data used to calculate overall hand-hygiene practices in livestock practice and equine practice reported by veterinarians (n=129)

	Livestock practice			Equine practice			Livestock vs. equine practice	
	n	%	95% CI	n	%	95% CI	p-value[a]	corrected p-value[b]
Washing hands between animals/animal groups								
Always	7	6.1	3.0–12.0	7	6.4	3.1–12.6	0.93	0.95
Often	41	35.7	27.5–44.7	28	25.5	18.2–34.3	0.10	0.23
Sometimes	25	21.7	15.2–30.1	35	31.8	23.9–41.0	0.09	0.22
Seldom	37	32.2	24.3–41.2	33	30.0	22.2–39.1	0.72	0.86
Never	5	4.3	1.9–9.8	7	6.4	3.1–12.6	0.48	0.66
Total	115			110				
Washing hands before moving on to next farm								
Always*	84	73.7	64.9–80.9	43	38.4	29.9–47.6	<0.001	<0.001
Often*	26	22.8	16.1–31.3	51	45.5	36.6–54.8	<0.001	<0.01
Sometimes	4	3.5	1.4–8.7	13	11.6	6.9–18.9	0.02	0.07
Seldom	0	0.0	0.0–3.3	3	2.7	0.9–7.6	0.08	0.21
Never	0	0.0	0.0–3.3	2	1.8	0.5–6.3	0.15	0.28
Total	114			112				
Use of hand sanitizer between animals/animal groups								
Always	4	3.5	1.4–8.7	1	0.9	0.2–4.7	0.18	0.32
Often	11	9.7	5.5–16.6	10	8.6	4.7–15.1	0.77	0.89
Sometimes	24	21.2	14.7–29.7	30	25.9	18.8–34.5	0.40	0.57
Seldom	32	28.3	20.8–37.2	35	30.2	22.6–39.1	0.75	0.88
Never	42	37.2	28.8–46.4	40	34.5	26.5–43.5	0.67	0.83
Total	113			116				
Use of hand sanitizer before moving on to next farm								

Always	16	14.7	9.2–22.5	18	15.9	10.3–23.8	0.80	0.91
Often	13	11.9	7.1–19.3	20	17.7	11.8–25.8	0.22	0.37
Sometimes	28	25.7	18.4–34.6	30	26.5	19.3–35.4	0.89	0.95
Seldom	27	24.8	17.6–33.6	29	25.7	18.5–34.4	0.88	0.95
Never	25	22.9	16.0–31.7	16	14.2	8.9–21.8	0.09	0.23
Total	109			113				

Abbreviation: CI, confidence interval

[a] z-test

[b] Benjamini-Hochberg false discovery rate correction

* Statistically significant at 5% level.

TABLE S5 Questions used for calculation of the overall precaution awareness (PA) score (all questions), PA score for livestock practice (questions on hygiene behaviour in livestock practice and general hygiene behaviour) and PA score for equine practice (questions on hygiene behaviour in equine practice and general hygiene behaviour)

Hygiene behaviour in livestock practice	Hygiene behaviour in equine practice	General hygiene behaviour
Hand-washing practices:		
Washing hands when dirty	Washing hands when dirty	
Overall hand hygiene between animals/animal groups	Overall hand hygiene between animals/animal groups	
Overall hand hygiene before moving on to next farm	Overall hand hygiene before moving on to next farm	
Use of hand sanitizer:		
Use of hand sanitizer after washing hands	Use of hand sanitizer after washing hands	Use of hand sanitizer after doffing protective gloves
Use of protective gear:		Frequency of cleaning/changing equipment:
Use of protective gloves when treating a contaminated, fresh wound in livestock	Use of protective gloves when treating a contaminated, fresh wound in horses	Protective jacket/coveralls
Use of protective gloves when treating an infected wound (signs of infection) in livestock	Use of protective gloves when treating an infected wound (signs of infection) in horses	Rubber boots
Wearing protective jacket/coveralls	Wearing protective jacket/coveralls	Stethoscope
Wearing work footwear	Wearing work footwear	

TABLE S6 Adequate hand-washing facilities (warm water, soap, fresh towel/paper hand towels) reported by respondents (n=129)

	On farms			In stables			Farms vs. stables	
	n	%	95% CI	n	%	95% CI	p-value[a]	corrected p-value[b]
Adequate hand-washing facilities								
Always*	13	11.0 %	6.5–17.9	1	0.9 %	0.2–4.7	<0.01	<0.01
Often*	79	66.9 %	58.0–74.8	25	21.4 %	14.9–29.6	<0.001	<0.001
Sometimes*	21	17.8 %	11.9–25.7	53	45.3 %	36.6–54.3	<0.001	<0.001
Seldom*	5	4.2 %	1.8–9.5	35	29.9 %	22.4–38.7	<0.001	<0.001
Never	0	0.0 %	0.0–3.2	3	2.6 %	0.0–3.2	0.08	0.08
Total	118			117				

Abbreviation: CI, confidence interval

[a] z-test

[b] Benjamini-Hochberg false discovery rate correction

* Statistically significant at 5% level.

TABLE S7 Use of hand sanitizer after doffing protective gloves as reported by web-based questionnaire respondents (n=126)

Use of hand sanitizer after doffing protective gloves	n	%	95% CI
Always	35	27.8	20.7–36.2
Often	53	42.1	33.8–50.8
Sometimes	21	16.7	11.2–24.1
Seldom	13	10.3	6.1–16.9
Never	4	3.2	1.2–7.9
Abbreviation: CI, confindence interval			

TABLE S8 Results of univariable logistic regression

	n	B	Wald's p-value	OR	95% CI
Overall precaution awareness (PA) score					
Years since graduation (three categories)	86		0.90		
<10 years	47	0.14	0.82	1.15	0.34–3.80
10–20 years	19	0.33	0.65	1.39	0.34–5.62
>20 years	20			Ref	
Years since graduation (four categories)	86		0.04		

	Not yet graduated	8	2.20	0.02	9.00	1.36–59.78
	<10 years	39	0.42	0.53	0.66	0.18–2.41
	10–20 years	19	0.33	0.65	1.39	0.34–5.62
	>20 years	20			Ref	
Age		71		0.56		
	26-31 years	27	0.57	0.38	1.77	0.49–6.34
	32-43 years	24	0.00	1.00	1.00	0.25–3.94
	44-85 years	20			Ref	
Gender		85				
	female	68	0.67	0.34	0.51	0.13–1.99
	male	17			Ref	
Precaution awareness (PA) score for livestock practice						
Years since graduation (three categories)		97		0.60		
	<10 years	50	0.54	0.36	1.71	0.54–5.42
	10–20 years	22	0.62	0.36	1.87	0.49–7.05
	>20 years	25			Ref	
Years since graduation (four categories)		97		0.54		
	Not yet graduated	9	1.16	0.16	3.20	0.62–16.49
	<10 years	41	0.38	0.53	1.47	0.44–4.87
	10–20 years	22	0.62	0.36	1.87	0.49–7.05
	>20 years	25			Ref	
Age		81		0.36		
	26-31 years	30	0.89	0.16	2.43	0.71–8.28
	32-43 years	25	0.68	0.30	1.98	0.54–7.16
	44-85 years	26			Ref	
Gender		96				
	female	76	-0.90	0.18	0.41	0.11–1.52
	male	20			Ref	
Precaution awareness (PA) score for equine practice						
Years since graduation (three categories)		90				
	<10 years	49	0.25	0.68	1.28	0.39–4.17
	10–20 years	20	0.07	0.93	1.07	0.26–4.44
	>20 years	21			Ref	
Years since graduation (four categories)		90		0.02		
	Not yet graduated	8	3.11	0.01	22.40	2.20–228.73
	<10 years	41	-0.42	0.53	0.66	0.18–2.40
	10–20 years	20	0.07	0.93	1.07	0.26–4.44
	>20 years	21			Ref	

Age		75		0.28		
	26-31 years	27	0.85	0.19	2.34	0.66–8.23
	32-43 years	26	0.02	0.98	1.02	0.26–3.94
	44-85 years	22			Ref	
Gender		89				
	female	72	-1.13	0.16	0.32	0.07–1.54
	male	17			Ref	

Abbreviations: B, beta coefficient; OR, odds ratio; CI, confidence interval; Ref, reference category

TABLE S9 Results of multivariable logistic regression with overall precaution awareness score as dependent variable (n=85)

		B	Wald's p-value	OR	95% CI
Years since graduation			0.04		
	Not yet graduated	1.99	0.04	7.33	1.06–50.67
	<10 years	−0.68	0.34	0.51	0.13–2.02
	10–20 years	−0.07	0.93	0.94	0.21–4.21
	>20 years			Ref	
Gender (female vs male)		−0.80	0.29	0.45	0.10–2.00
Constant		−0.78	0.19	0.46	

Note: Cox & Snell R^2 = 0.12, Nagelkerke R^2 = 0.18, Hosmer and Lemeshow goodness of fit statistic = 1.35 (p-value = 0.85).

Abbreviations: B, beta coefficient; OR, odds ratio; CI, confidence interval; Ref, reference category

TABLE S10 Results of multivariable logistic regression with precaution awareness score for equine practice as the dependent variable (n=87)

		B	Wald's p-value	OR	95% CI
Years since graduation			0.02		
	Not yet graduated	2.90	0.02	18.19	1.67–198.79
	<10 years	−0.82	0.24	0.44	0.11–1.74
	10–20 years	−0.50	0.53	0.61	0.13–2.90
	>20 years			Ref	
Gender (female vs male)		−1.49	0.11	0.23	0.04–1.40
Constant		−0.67	0.25	0.51	

Note: Cox & Snell R^2 = 0.19, Nagelkerke R^2 = 0.28, Hosmer and Lemeshow goodness of fit statistic = 0.44 (p-value = 0.98).

Abbreviations: B, beta coefficient; OR, odds ratio; CI, confidence interval; Ref, reference category

TABLE S11 Results of multivariable logistic regression with precaution awareness score for equine practice as the dependent variable excluding outliers (standardized residuals >2.5) (n=85)

	B	Wald's p-value	OR	95% CI
Years since graduation		0.78		
Not yet graduated	39.40	1.00	$1.29e^{17}$	0.00–
<10 years	−0.76	0.30	0.47	0
10–20 years	−0.41	0.63	0.67	0.11–2.00
>20 years			Ref	
Gender (female vs male)	−19.83	1.00	0.00	0.00–
Constant	−0.69	0.26	0.50	

Note: Cox & Snell R^2 = 0.29, Nagelkerke R^2 = 0.42, Hosmer and Lemeshow goodness of fit statistic = 0.00 (p-value = 1000.00).

Abbreviations: B, beta coefficient; OR, odds ratio; CI, confidence interval; Ref, reference category
