# Supplementary material for: Infection prevention and control practices of ambulatory veterinarians: A questionnaire study in Finland
Source: Vet Med Sci. 2021 Mar 1;7(4):1059–70. doi: 10.1002/vms3.464 (PMC8294370; doi:10.1002/vms3.464)
Supplement: Supplementary file 2 — Supplementary Material [file VMS3-7-1059-s001.pdf]

HELSINGIN YLIOPISTO
HELSINGFORS UNIVERSITET
UNIVERSITY OF HELSINKI

Moniresistentit bakteerit eläinlääkäreillä

1. NÄYTEKOODI

Näytekoodilla tarkoitetaan suostumuslomakkeeseen, näytepurkkiin ja vanutikkuun merkittyä koodia.

*** Näytekoodi**

*** Näytekoodi uudelleen (*Tarkista, että koodit täsmäävät.*)**

2. NÄYTTEENANTAJAN PERUSTIEDOT

Sukupuoli

Valitse:
- Mies
- Nainen
- Muu

Syntymävuosi

-- Valitse tästä --

Pääasiallinen sijoittuminen työelämään viimeisen 12 kk:n aikana

Valitse:
- Sijaisuuksia tekevä (määräaikaisia työsuhteita)
- Kunta
- Yksityisessä praktiikassa työntekijänä
- Yrittäjä tai ammatinharjoittaja
- Valtio tai EU
- Yliopisto
- Yksityisellä sektorilla työntekijänä (teollisuudessa, lääke-alan yrityksissä, järjestöissä tai liike-elämässä)
- Opiskelija
- Eläkeläinen
- Tilapäisesti työelämän ulkopuolella
- Muu, mikä ?

Jos muu, mikä?

ELL-tutkinnon (tai vastaavantasoisen tutkinnon) suorituspaikka

* Yliopisto/ korkeakoulu --Valitse tästä--

Valmistumisvuosi (jos olet valmistunut) -- Valitse tästä --

3. TYÖOLOSUHTEET

Opintoihin liittyvät eläinkontaktit rinnastetaan seuraavissa kysymyksissä työperäisiin eläinkontakteihin.

Oletko työssäsi viimeisten 12 kk:n aikana ollut kontaktissa:

	Kyllä olen	En ole
* eläviin eläimiin	○	○
* eläinten ruhoihin tai raatoihin	○	○
* eläinperäisiin näytteisiin	○	○

Työnkuva viimeisen 12 kk:n aikana
On tärkeää, että vastaat jokaiseen kohtaan.

	Vähintään viikottain	Harvemmin	Ei lainkaan
* Nautapraktiikkaa	○	○	○
* Sikapraktiikkaa	○	○	○
* Siipikarjapraktiikkaa	○	○	○
* Turkiseläinpraktiikkaa	○	○	○
* Muuta tuotantoeläinpraktiikkaa	○	○	○
* Kiertävää hevospraktiikkaa	○	○	○
* Hevosklinikalla	○	○	○
* Pieneläinpraktiikkaa vastaanotolla/klinikalla	○	○	○
* Pieneläinpraktiikkaa sairaalassa (potilaita yön yli)	○	○	○
* Maatilakäyntejä (ei praktiikkaan liittyviä)	○	○	○
* Eläinsuojelukäyntejä	○	○	○
* Raadonavauksia	○	○	○
* Teurastamolla	○	○	○
* Laboratoriossa	○	○	○
* Opetus- ja/tai tutkimustyötä	○	○	○
* Toimistotyössä	○	○	○
* Muu työnkuva, mikä?	○	○	○

Jos muuta tuotantoeläinpraktiikkaa tai muita työnkuvia, tarkenna tähän

Jos et ole työssäsi ollut viimeisen 12 kk:n aikana lainkaan kontaktissa eläimiin, niiden raatoihin tai eläinperäisiin näytteisiin, voit siirtyä sivulle 5, kohtaan 5, Altistuminen vapaa-ajalla.

Minne eläinkontaktit (viimeisen 12kk:n aikana) sijoittuivat?
Voit valita useita.

- ☐ Etelä-Suomi
- ☐ Itä-Suomi
- ☐ Lappi
- ☐ Lounais-Suomi
- ☐ Pohjois-Suomi
- ☐ Länsi- ja Sisä-Suomi
- ☐ Ahvenanmaa
- ☐ Ulkomaille, minne?

Jos vastasit ulkomaille, minne?

☐ **En ole viimeisen 12 kk:n aika tehnyt lainkaan praktiikkaa**

Jos et ole viimeisen 12 kk:n aikana tehnyt lainkaan praktiikkaa, voit siirtyä sivulle 4, kohtaan 4, Työkäytännöt.

Jos työskentelit pieneläin-, hevos- tai tuotantoeläinpraktiikassa viimeisen 12kk:n aikana, työskentelitkö pääasiallisesti:

	Ainoana eläinlääkärinä	Yhteisvastaanotolla	Muu järjestely, mikä?	Ei koske minua	Jos muu, tarkenna:
Valitse:	○	○	○	○	

Työskentelikö vastaanotolla/klinikalla samanaikaisesti kanssasi hoitaja/ hoitajia?

	Täyspäiväisesti	Osa-aikaisesti	Ei työskennellyt
Valitse:	○	○	○

☐ **En ole viimeisen 12 kk:n aikana tehnyt lainkaan pieneläinpraktiikkaa**

Jos et ole viimeisen 12 kk:n aikana tehnyt lainkaan pieneläinpraktiikkaa, voit siirtyä sivulle 4, kohtaan 4, Työkäytännöt.

Lemmikkieläinkontaktit työssä viimeisen 12kk:n aikana
On tärkeää, että vastaat jokaiseen kohtaan.

	Vähintään viikottain	Harvemmin	Ei lainkaan
Koira	○	○	○
Kissa	○	○	○
Kani	○	○	○
Jyrsijä	○	○	○
Matelija	○	○	○
Minisika	○	○	○
Lemmikkilintu	○	○	○
Muu, mikä?	○	○	○

Jos vastasit muu, tarkenna mitä eläimiä.

4. TYÖKÄYTÄNNÖT

Jos et työssäsi ole lainkaan ollut kontaktissa eläimiin, niiden raatoihin tai eläinperäisiin näytteisiin, voit siirtyä sivulle 5, kohtaan 5, Altistuminen vapaa-ajalla.

Olivatko (viimeisen 12kk:n aikana) käsienpesumahdollisuutesi riittävät (lämmin vesi, saippua, puhdas pyyhe/käsipyyhe) seuraavissa yhteyksissä?
On tärkeää, että vastaat jokaiseen kohtaan.

	Aina	Usein	Joskus	Harvoin	Ei koskaan	Ei koske minua
Tuotantoeläintilat						
Tallit						
Hevosklinikka						
Pieneläinvastaanotto/ -klinikka/ -sairaala						
Teurastamo						
Laboratorio						
Muu, mikä?						

Jos muu, mikä?

Pystyitkö (viimeisen 12kk:n aikana) pesemään kätesi:
On tärkeää, että vastaat jokaiseen kohtaan.

	Ennen eläinkontaktia/ kontaktia eläinperäiseen näytteeseen/ raatoon?						Eläin-, näyte- tai raatokontaktin jälkeen?					
	Aina	Usein	Joskus	Harvoin	Ei koskaan	Ei koske minua	Aina	Usein	Joskus	Harvoin	Ei koskaan	Ei koske minua
Tuotantoeläintilat												
Tallit												
Hevosklinikka												
Pieneläinvastaanotto/-klinikka/ -sairaala												
Teurastamo												
Laboratorio												
Muu, mikä?												

Jos muu työympäristö, mikä?

Eläinten kanssa työssäni toimiessani pesin käteni (viimeisen 12kk:n aikana)
On tärkeää, että vastaat jokaiseen kohtaan.

	Kun käteni olivat likaiset						Eläinten/eläinryhmien välissä						Ennen seuraavalle tilalle siirtymistä (kiertävä praktiikka/ tilakäynnit)					
	Aina	Usein	Joskus	Harvoin	Ei koskaan	Ei koske minua	Aina	Usein	Joskus	Harvoin	Ei koskaan	Ei koske minua	Aina	Usein	Joskus	Harvoin	Ei koskaan	Ei koske minua
Tuotantoeläimet																		
Hevonen																		
Lemmikkieläimet																		
Muu eläin, mikä?																		

Jos muu eläin, mikä?

Kuinka kauan pesit (viimeisen 12 kk:n aikana) käsiäsi eläinkontaktien yhteydessä? *(Keskimääräinen aika sekunneissa, jonka käytit käsien hieromiseen saippualla)* [ ]

Eläinten kanssa työssäni toimiessani käytin desinfiointihuuhdetta (viimeisen 12 kk:n aikana)
On tärkeää, että vastaat jokaiseen kohtaan.

	Käsien pesun jälkeen						Eläinten/eläinryhmien välissä						Ennen seuraavalle tilalle siirtymistä (kiertävä praktiikka/ tilakäynnit)					
	Aina	Usein	Joskus	Harvoin	Ei koskaan	Ei koske minua	Aina	Usein	Joskus	Harvoin	Ei koskaan	Ei koske minua	Aina	Usein	Joskus	Harvoin	Ei koskaan	Ei koske minua
Tuotantoeläimet	○	○	○	○	○	○	○	○	○	○	○	○	○	○	○	○	○	○
Hevonen	○	○	○	○	○	○	○	○	○	○	○	○	○	○	○	○	○	○
Lemmikkieläimet	○	○	○	○	○	○	○	○	○	○	○	○	○	○	○	○	○	○
Muu eläin, mikä?	○	○	○	○	○	○	○	○	○	○	○	○	○	○	○	○	○	○

Jos muu eläin, mikä?

Mitä suojavaatteita käytit toimiessasi työssäsi (poislukien kirurgia, hammashoito) seuraavien eläinten kanssa (viimeisen 12 kk:n aikana)?
On tärkeää, että vastaat jokaiseen kohtaan.

	Työtakki/haalarit						Työjalkineet						Päähine					
	Aina	Usein	Joskus	Harvoin	Ei koskaan	Ei koske minua	Aina	Usein	Joskus	Harvoin	Ei koskaan	Ei koske minua	Aina	Usein	Joskus	Harvoin	Ei koskaan	Ei koske minua
Tuotantoeläimet	○	○	○	○	○	○	○	○	○	○	○	○	○	○	○	○	○	○
Hevonen	○	○	○	○	○	○	○	○	○	○	○	○	○	○	○	○	○	○
Lemmikkieläimet	○	○	○	○	○	○	○	○	○	○	○	○	○	○	○	○	○	○
Muu eläin, mikä?	○	○	○	○	○	○	○	○	○	○	○	○	○	○	○	○	○	○

Jos muu eläin, mikä?

Käytin suojakäsineitä toimiessani työssäni (poislukien kirurgia, hammashoito) seuraavien eläinten kanssa (viimeisen 12kk:n aikana):
On tärkeää, että vastaat jokaiseen kohtaan.

	Aina	Usein	Joskus	Harvoin	Ei koskaan	Ei koske minua
Tuotantoeläimet	○	○	○	○	○	○
Hevonen	○	○	○	○	○	○
Lemmikkieläimet	○	○	○	○	○	○
Muu, mikä?	○	○	○	○	○	○

Jos muu eläin, mikä?

Käytin suojakäsineitä seuraavissa toimenpiteissä seuraavilla eläimillä (viimeisen 12 kk:n aikana)
On tärkeää, että vastaat jokaiseen kohtaan.

	Likaisen, tuoreen haavan hoito						Infektoituneen haavan hoito (tulehdusmerkit)					
	Aina	Usein	Joskus	Harvoin	Ei koskaan	Ei koske minua	Aina	Usein	Joskus	Harvoin	Ei koskaan	Ei koske minua
Tuotantoeläimet												
Hevonen												
Lemmikkieläimet												
Muu, mikä?												

Jos muu eläin tai muu tuotantoeläin, mikä?

Käyttäessäni suojakäsineitä eläinten kanssa toimiessani pesin ja/tai desinfioin käteni (viimeisen 12 kk:n aikana):
On tärkeää, että vastaat jokaiseen kohtaan.

	Aina	Usein	Joskus	Harvoin	Ei koskaan	Ei koske minua
Ennen käsineiden laittamista						
Käsineiden riisumisen jälkeen						

Käytin hengityssuojainta toimiessani työssäni (poislukien kirurgia, hammashoito) seuraavien eläinten kanssa (viimeisen 12kk:n aikana):
On tärkeää, että vastaat jokaiseen kohtaan.

	Aina	Usein	Joskus	Harvoin	Ei koskaan	Ei koske minua
Nauta						
Sika						
Siipikarja						
Turkiseläimet						
Muut tuotantoeläimet						
Hevonen						
Muut tuotantoeläimet						
Lemmikkieläimet						
Muu, mikä?						
Muu, mikä?						

Jos muu eläin, mikä?

Jos et ole viimeisen 12 kk:n aikana tehnyt lainkaan praktiikkaa, voit siirtyä sivulle 5, kohtaan 5, Altistuminen vapaa-ajalla.

Kuinka usein puhdistit tai vaihdoit puhtaisiin seuraavat kiertävään praktiikkaan liittyvät työvälineesi (viimeisen 12 kk:n aikana)?
Rinnasta tilojen tarjoamat varusteet omiin varusteisiisi. On tärkeää, että vastaat jokaiseen kysymykseen.

	Eläinten/eläinryhmien välissä	Tilakäyntien välissä	Kerran päivässä	Viikottain, muttei päivittäin	Harvemmin kuin kerran viikossa	Ei koske minua
Suojatakki/-haalarit (myös asiakkaan tarjoamat)						
Kumisaappaat (myös asiakkaan tarjoamat)						
Turvakengät (myös asiakkaan tarjoamat)						
Praktiikkalaukku (ulkoa)						
Praktiikkalaukku (sisältö)						

Stetoskooppi	○	○	○	○	○	○
Muut tilakäynnillä käyttämäni, eläinkontaktissa olleet välineet	○	○	○	○	○	○

Tarkenna halutessasi tähän

Kuinka usein puhdistit tai vaihdoit puhtaisiin seuraavat vastaanotolla/ klinikalla käyttämäsi työvälineet (viimeisen 12 kk:n aikana)?
On tärkeää, että vastaat jokaiseen kohtaan.

	Päivittäin	Viikottain, muttei päivittäin	Harvemmin kuin kerran viikossa	Ei koske minua
Työvaatteet	○	○	○	○
Työsandaalit	○	○	○	○
Turvakengät	○	○	○	○
Stetoskooppi	○	○	○	○
Vastaanoton (ei-steriilit) instrumentit	○	○	○	○

Tarkenna halutessasi tähän

5. ALTISTUMINEN VAPAA-AJALLA

Asuitko eläintilalla (viimeisen 12 kk:n aikana)? Jos asuit, tarkenna mitä eläimiä tilalla pidetään.

	Asuin	En asunut	Tarkenna / kommentoi:
Valitse:	○	○	

Kuuluiko joku taloutesi muista aikuisista johonkin seuraavista ammattikunnista (viimeisen 12 kk:n aikana)?
Voit valita useita.

- ☐ Hoitotyö (esim. lääkäri, hammaslääkäri, hoitaja)
- ☐ Eläinlääkintä (eläinlääkäri, eläintenhoitaja)
- ☐ Maanviljelijä tai muu ammatti eläinten parissa
- ☐ Ei kuulunut

Mille seuraavista alueista olet matkustanut viimeisen 12 kk:n aikana?
Voit valita useita.

- ☐ Pohjoismaat
- ☐ Baltian maat
- ☐ Keski-Eurooppa (Itävalta, Belgia, Ranska, Saksa, Liechtenstein, Luxemburg, Monaco, Alankomaat, Sveitsi, Iso-Britannia, Irlanti)
- ☐ Etelä-Eurooppa (Portugali, Espanja, Gibraltar, Andorra, Italia, San Marino, Vatikaanivaltio, Malta, Slovenia, Kroatia, Bosnia ja Hertsegovina, Montenegro, Serbia, Albania, Makedonia ja Kreikka)
- ☐ Itä-Eurooppa (Valkovenäjä, Bulgaria, Tsekki, Unkari, Puola, Moldova, Romania,Venäjä, Slovakia, Ukraina)
- ☐ Aasia
- ☐ Pohjois-Amerikka
- ☐ Väli-Amerikka
- ☐ Etelä-Amerikka
- ☐ Afrikka
- ☐ Australia ja Oseania
- ☐ en ole matkustanut Suomen ulkopuolella

☐ **En ole vapaa-ajallani ollut kontaktissa eläimiin viimeisen 12 kk:n aikaina**

Jos et viimeisen 12 kk aikana ole ollut vapaa-ajallasi kontaktissa eläimiin, voit siirtyä sivulle 6, kohtaan 6, Oma terveydentila.

Kuinka usein olet viimeisen 12 kk:n aikana vapaa-ajallasi tai kotonasi ollut kontaktissa seuraaviin eläimiin?
On tärkeää, että vastaat jokaiseen kohtaan.

	Vähintään viikottain	Harvemmin	Ei lainkaan
Nauta	○	○	○
Sika	○	○	○
Siipikarja	○	○	○
Turkiseläimet	○	○	○
Muut tuotantoeläimet	○	○	○
Hevonen	○	○	○
Koira	○	○	○
Kissa	○	○	○
Kani	○	○	○
Jyrsijä	○	○	○
Matelija	○	○	○
Lemmikkilintu	○	○	○
Muu, mikä?	○	○	○

Jos muu, mikä?

6. TERVEYDENHUOLTOON LIITTYVÄT RISKIT

Oletko käynyt lääkärissä viimeisen 12 kk:n aikana?

	Olen	En ole
Valitse:	○	○

Missä olet käynyt lääkärissä viimeisen 12 kk:n aikana?
Voit valita useita vaihtoehtoja.

- ☐ Työterveyshuolto (kotimaassa)
- ☐ Muu avoterveydenhoito (kotimaassa)
- ☐ Avoterveydenhoito (ulkomailla)
- ☐ Sairaalahoito (kotimaassa)
- ☐ Sairaalahoito (ulkomailla)

Onko sinulla jokin lääkärin hoitoa vaativa, immuunipuolustusta heikentävä pitkäaikaissairaus?

	Kyllä on	Ei ole
Valitse:	○	○

Onko sinulla immunosupressiivinen lääkitys? (esim. systeeminen kortisoni > 1kk, biologinen lääke tai sytostaatti)?

	Kyllä on	Ei ole
Valitse:	○	○

Oletko ollut antibioottikuurilla?

	Viimeisen kuukauden aikana		Viimeisen 12 kk:n aikana	
	Kyllä	En	Kyllä	En
Valitse:	○	○	○	○

Jos vastasit kyllä, mitä antibioottia/antibiootteja?

Jos aloitit kuurin ulkomailla, missä maassa?

Tiedätkö kantavasi tai kantaneesi joskus jotain mikrobilääkkeille resistenttiä bakteeria (esim. MRSA, ESBL)?

	Kyllä	En
Valitse:	○	○

Jos vastasit kyllä, tarkenna halutessasi tähän (mikä bakteeri, ajankohta, tiedätkö puhdistuneesi).

Jos vastasit kyllä, oliko kyseessä mikrobilääkkeille resistentin bakteerin aiheuttama infektio vai kolonisaatio? ?

	Infektio	Kolonisaatio	Ei koske minua
Valitse:	○	○	○

7. ZOONOOSITIETÄMYS

Minulla on hyvät tiedot zoonooseista ja niiden ehkäisystä.

	Täysin eri mieltä	Eri mieltä	Osittain eri mieltä	Ei mielipidettä	Osittain samaa mieltä	Samaa mieltä	Täysin samaa mieltä
Valitse:	○	○	○	○	○	○	○

Zoonoosit voivat tarttua:
Voit valita useita.

- ☐ Eläimistä ihmisiin
- ☐ Ihmisistä eläimiin
- ☐ Välillisesti
- ☐ Suorassa kontaktissa

8. PALAUTE

Lämmin kiitos vastauksistasi! Voit halutessasi antaa palautetta kyselystä alla olevaan kenttään.

TIETOJEN LÄHETYS

Tallenna Esitäyttö URL
